# Supplementary material for: Toughening self-healing elastomer crosslinked by metal–ligand coordination through mixed counter anion dynamics
Source: Nat Commun. 2023 Aug 18;14:5026. doi: 10.1038/s41467-023-40791-z (PMC10439188; doi:10.1038/s41467-023-40791-z)
Supplement: Supplementary file 1 — Supplementary Information [file 41467_2023_40791_MOESM1_ESM.pdf]

**Supplementary Information**

# Toughening self-healing elastomer crosslinked by metal–ligand coordination through mixed counter anion dynamics

Hyunchang Park,[1,2] Taewon Kang,[1] Hyunjun Kim,[1] Jeong-Chul Kim,[3] Zhenan Bao[2] and Jiheong Kang[1]*

[1] Department of Materials Science and Engineering, Korea Advanced Institute of Science and Technology (KAIST), Daejeon, 34141 Republic of Korea

[2] Department of Chemical Engineering, Stanford University, Stanford, California 94305, United States

[3] Center for Nanomaterials and Chemical Reactions, Institute for Basic Science (IBS), Daejeon, 34141 Republic of Korea

* To whom correspondence should be addressed:

E-mail: jiheongkang@kaist.ac.kr (J. K.)

# Supplementary Methods

## Materials and general measurements

The chemicals purchased are used as received without further purification. 2,2'-bipyridine-5,5'-dicarboxylic acid (97%), thionyl chloride (97%), triethylamine (≥99.5%), zinc chloride (≥99.995%), zinc acetate (99.99%), zinc trifluoromethanesulfonate (98%), zinc di[bis(trifluoromethylsulfonyl)imide] (95%), copper(II) chloride (99.999%), and copper(II) trifluoromethanesulfonate (98%) were purchased from Sigma-Aldrich. Bis(3-aminopropyl) terminated poly(dimethylsiloxane) ($H_2N$–PDMS–$NH_2$, $M_n$ = 5 kDa) (95–100%) macromonomers were purchased from Gelest. Bis(2,4-pentanedionato)zinc(II) ($Zn(acac)_2$) (>96.0%) was purchased from TCI. Bis(2,4-pentanedionato)copper(II) ($Cu(acac)_2$) (98%) was purchased from Alfa Aesar. The solvent DCM was saturated with argon and purified by passage through activated $Al_2O_3$ columns under argon (Chembly SPS H4). All air-sensitive manipulations were carried out under an argon atmosphere by standard Schlenk-line techniques. Solution $^1$H NMR spectra were recorded on a Bruker Avance NEO 400 (400 MHz) spectrometer. Chemical shifts were referenced to the residual non-deuterated $CHCl_3$ solvent peak (δ 7.26 ppm). Size exclusion chromatography (SEC) traces were obtained using a Viscotek TDA302 instrument equipped with a RI detector and packing column (three PLgel 10 μm MIXED-B) using tetrahydrofuran (THF) as the eluent. The number and weight average molecular weights of the polymers were calculated relative to linear polystyrene standards. UV-vis spectra were recorded on a Jasco V-770 spectrophotometer. Optical microscope (OM) images were obtained on a Nikon ECLIPSE LV100N POL.

**Synthesis of BPy-PDMS.** BPy-PDMS polymer was prepared according to the literature procedure with slight modifications.[1,2] A flame-dried 250 mL 2-neck round bottom flask was charged with 2,2'-bipyridine-5,5'-dicarboxylic acid (1.15 g, 4.72 mmol) and thionyl chloride (70 mL) under Ar atmosphere. The mixture was heated at reflux for 12 h with vigorous stirring. After cooled to r.t., the volatile fractions of the crude mixture were removed thoroughly under reduced pressure to afford 2,2'-bipyridine-5,5'-dicarbonyl dichloride as a light-yellow solid. The crude product was used in the next polymerization reaction without further purification. The crude product and dry DCM (80 mL) were transferred to a flame-dried 250 mL 3-neck round bottom flask under an Ar atmosphere. The suspension was cooled to 0 °C, and a DCM (40 mL) solution of $H_2N$–PDMS–$NH_2$ (23.6 g, ~4.72 mmol) was added dropwise via syringe over a period of 30 min. To the reaction mixture was added dry triethylamine (1.5 mL). Then, the mixture was slowly warmed up to r.t., stirred for 1 d, and concentrated under reduced pressure. The resulting viscous mixture was dissolved in a minimum amount of chloroform (~50 mL), and an excess amount of MeOH (~300 mL) was added to induce precipitation. After 30 min, the liquid fraction was removed by decantation. This dissolution–precipitation–decantation process was repeated two more times, then the polymer was entirely dried under reduced pressure for 24 h at 40 °C to afford BPy-PDMS (19.4 g). $^1$H NMR of the repeating unit (400 MHz, $CDCl_3$, 298 K): δ 9.04 (s, 2H), δ 8.52 (d, $J$ = 9 Hz, 2H), δ 8.21 (d, $J$ = 8 Hz, 2H), δ 6.33 (br s, 2H), δ 3.50 (m, 4H), δ 1.70 (m, 4H), δ 0.63 (m, 4H), δ 0.07 (br s, 418H). $M_n$ = 43300, $M_w$ = 77700, Đ = 1.8.

**Method for metal–BPy crosslinking.** According to the $^1$H NMR proton integration values, the molecular

weight of the repeating unit of BPy-PDMS was estimated to be 5500. To a chloroform (15 mL) solution of BPy-PDMS (500 mg) was added a 150 μL aliquot of a MeOH solution (0.2 M) of $MX_2$ (M; $Zn^{2+}$ or $Cu^{2+}$, X; $acac^-$, $OAc^-$, $Cl^-$, $OTf^-$, $TFSI^-$). For the stock solutions of $Zn(acac)_2$ and $Zn(OAc)_2$, a tiny amount of acetic acid (1% v/v) was additionally added to completely dissolve the metal salt. To prepare the mixed anion samples 75 μL aliquots of each stock solution were added simultaneously. The reaction solution was stirred at r.t. for 20 h, poured into a Teflon mold, and dried overnight in the fume hood to furnish the crosslinked polymer as a freestanding film. The obtained film was further dried for 12 h under reduced pressure at 50 °C and annealed for 3h at 90 °C before use.

## Supplementary Figures

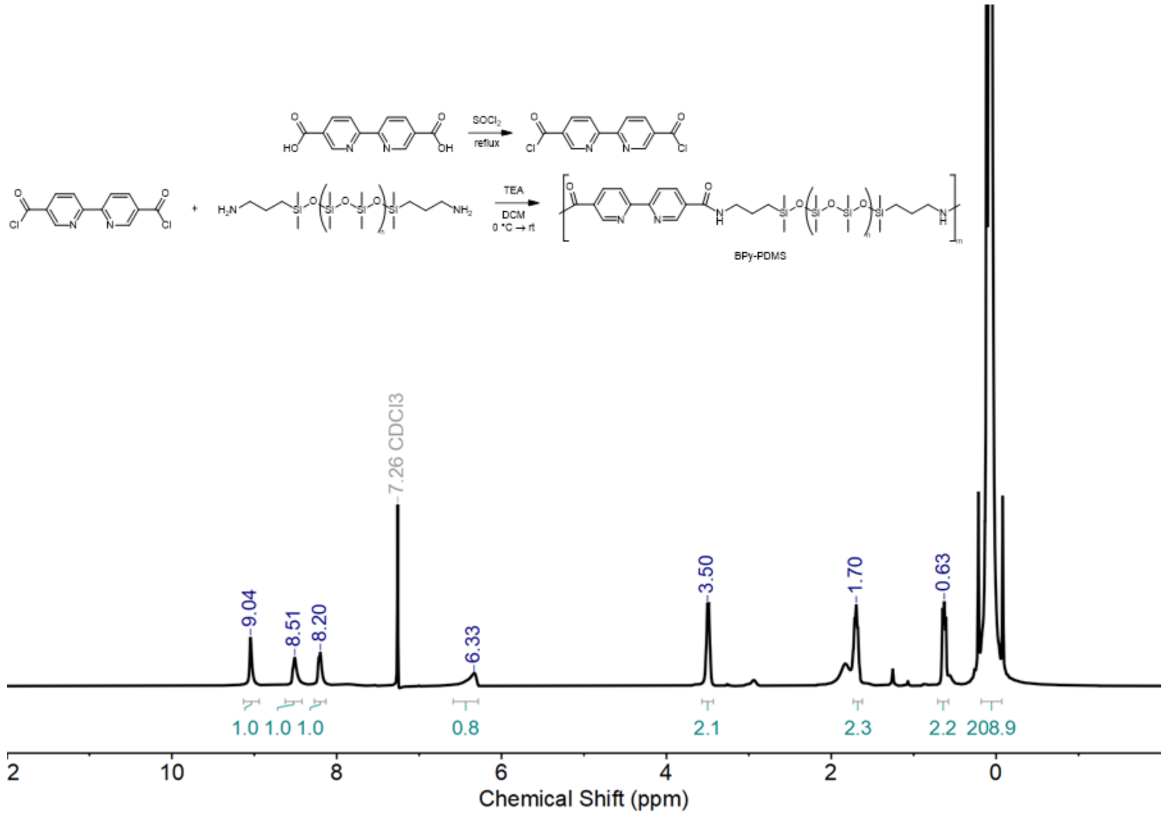


**Supplementary Fig. 1 | Synthesis and characterization of BPy-PDMS.** $^{1}$H NMR spectrum (400 MHz) of BPy-PDMS in $CDCl_3$ ($T$ = 298 K). Inset: synthetic route for BPy-PDMS.

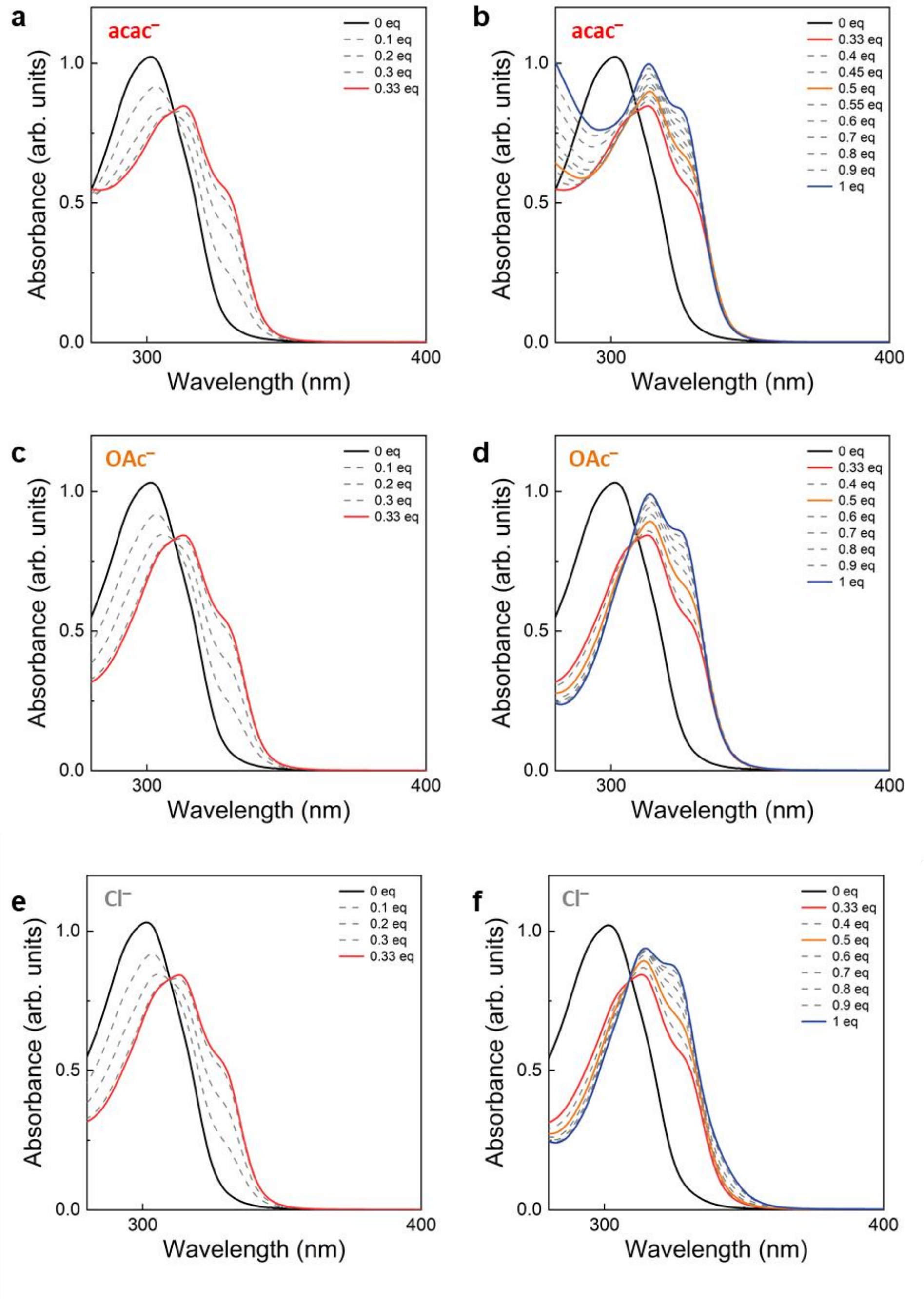


**Supplementary Fig. 2 | Titration analysis regarding coordinating anions.** UV-vis absorption spectra obtained by titration of a $CHCl_3$ solution of BPy-PDMS (33 μM) with $Zn^{2+}$ salt solutions of the coordinating counter anions. **a**, **b**, $Zn(acac)_2$, **c**, **d**, $Zn(OAc)_2$, **e**, **f**, $ZnCl_2$.

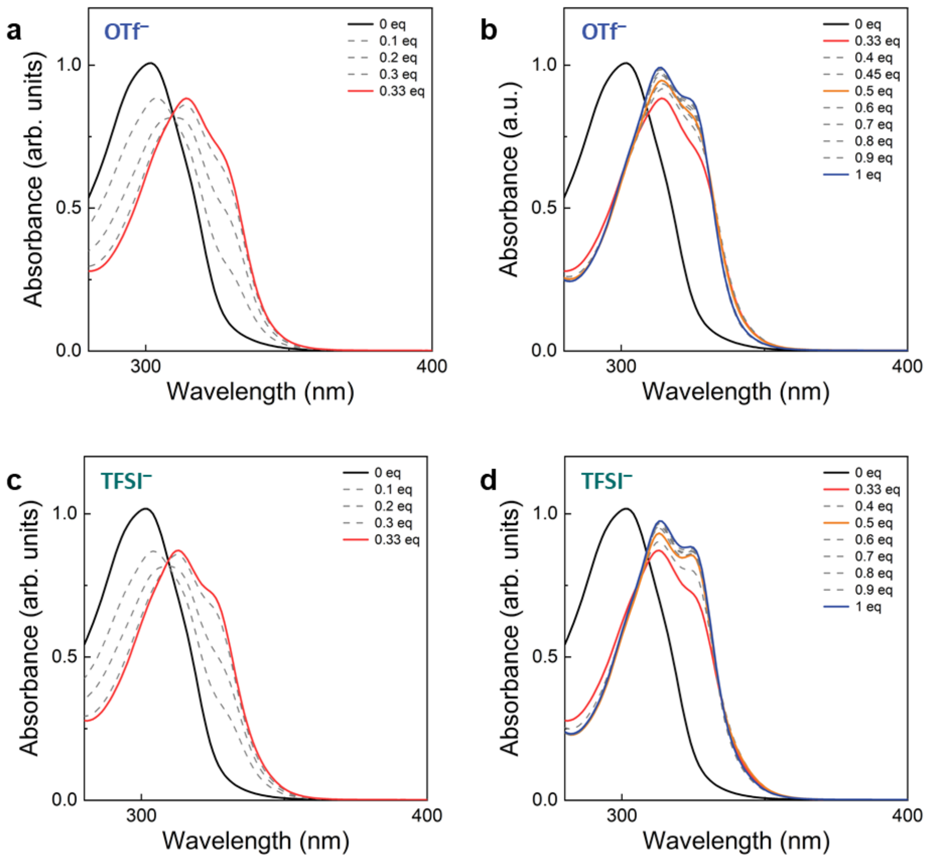


**Supplementary Fig. 3 | Titration analysis regarding non-coordinating anions.** UV-vis absorption spectra obtained by titration of a $CHCl_3$ solution of BPy-PDMS (33 μM) with $Zn^{2+}$ salt solutions of non-coordinating counter anions. **a**, **b**, $Zn(OTf)_2$, **c**, **d**, $Zn(TFSI)_2$.

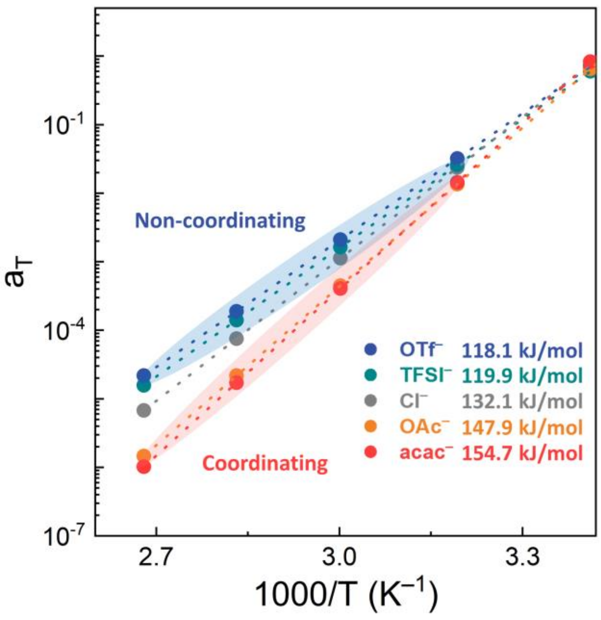


**Supplementary Fig. 4 | Arrhenius plots.** Arrhenius plots of the horizontal shift factor, $a_T$, of Zn-X-BPy-PDMS polymers (*T* range; 0–100 °C, $T_0$; 20 °C). Inset: calculated activation energy of each polymer for flow transition.

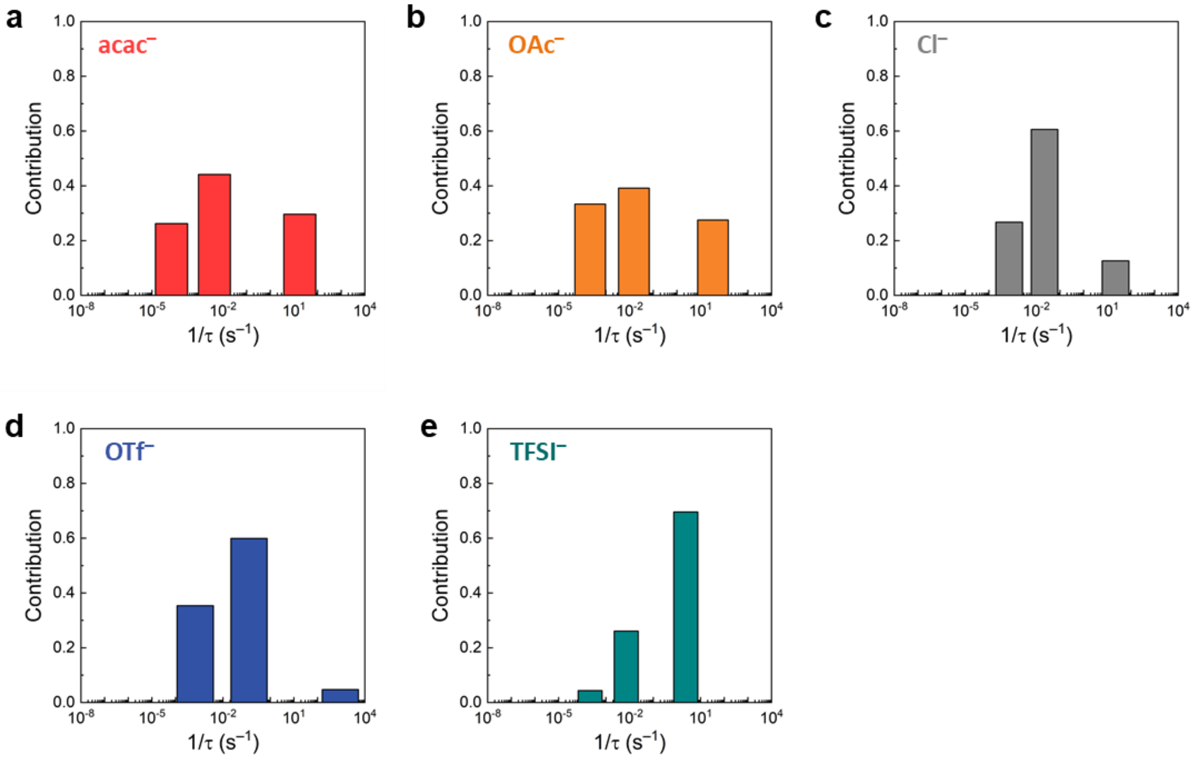


**Supplementary Fig. 5 | Contributions of each relaxation component to overall energy dissipation of Zn-X-BPy-PDMS polymers.** Relative contributions of each relaxation component of **a**, Zn-acac-BPy-PDMS, **b**, Zn-OAc-BPy-PDMS, **c**, Zn-Cl-BPy-PDMS, **d**, Zn-OTf-BPy-PDMS, and **e**, Zn-TFSI-BPy-PDMS. The sum of the three contributions equals to 1 for all the polymers.

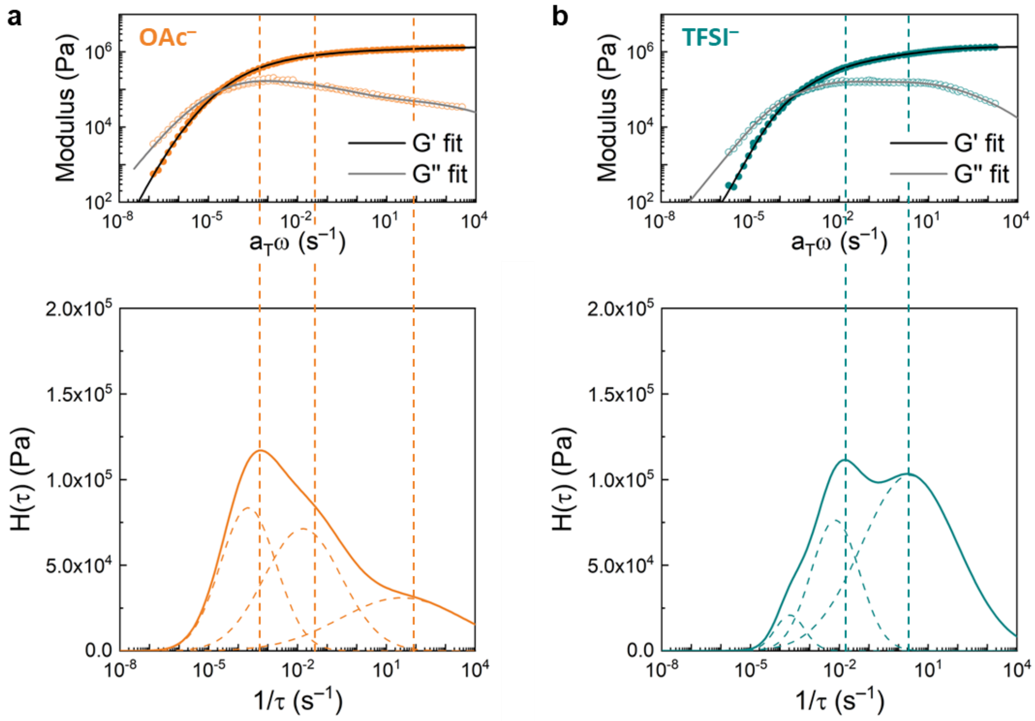


**Supplementary Fig. 6 | Rheological analysis of Zn-OAc-BPy-PDMS and Zn-TFSI-BPy-PDMS. a, b, top**, Experimental master curves (dots) and best-fit lines (G'; black line, G"; gray line) of **a**, Zn-OAc-BPy-PDMS, **b**, Zn-TFSI-BPy-PDMS. **a, b, bottom**, Relaxation time spectra, H($\tau$) of **a**, Zn-OAc-BPy-PDMS, **b**, Zn-TFSI-BPy-PDMS. The dashed lines in the plot indicate the spectra of each energy dissipation mode.

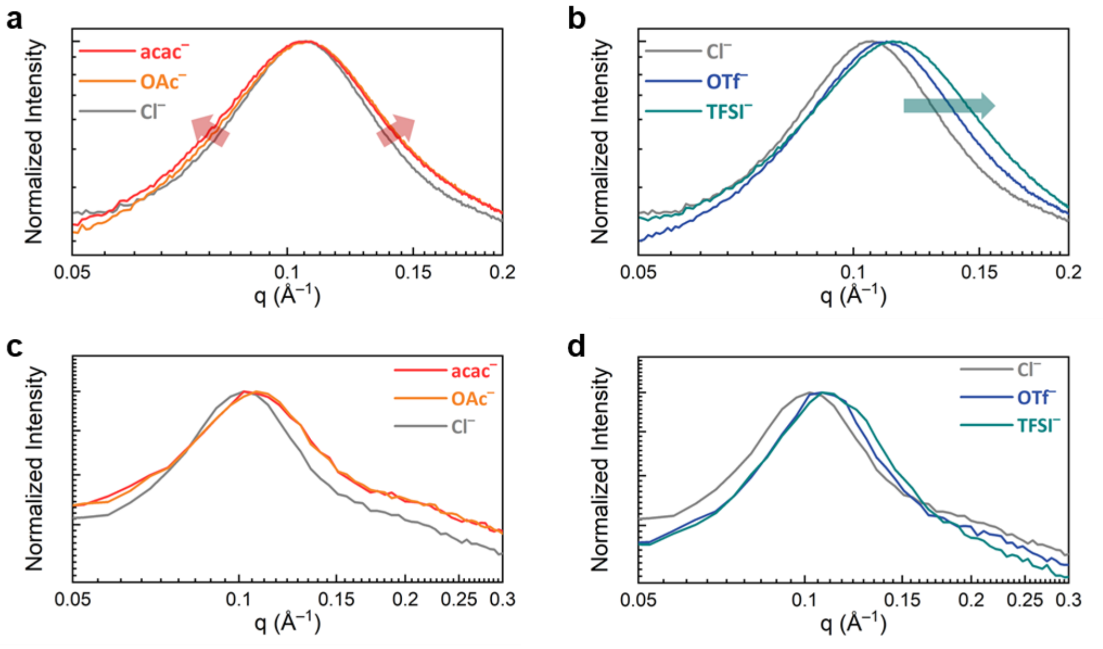


**Supplementary Fig. 7 | SAXS and SANS analysis of Zn-X-BPy-PDMS polymers.** Anion-dependent film SAXS (**a**, **b**) and SANS (**c**, **d**) profiles. **a**, **c**, Comparison between coordinating ($Cl^-$) and multimodal ($acac^-$ and $OAc^-$) anions. A broad scattering peak was observed in the profile of the multimodal anion. **b**, **d**, Comparison between coordinating ($Cl^-$) and non-coordinating ($OTf^-$ and $TFSI^-$) anions. The scattering peaks of the non-coordinating anions appear in the higher *q* region compared to the coordinating anion.

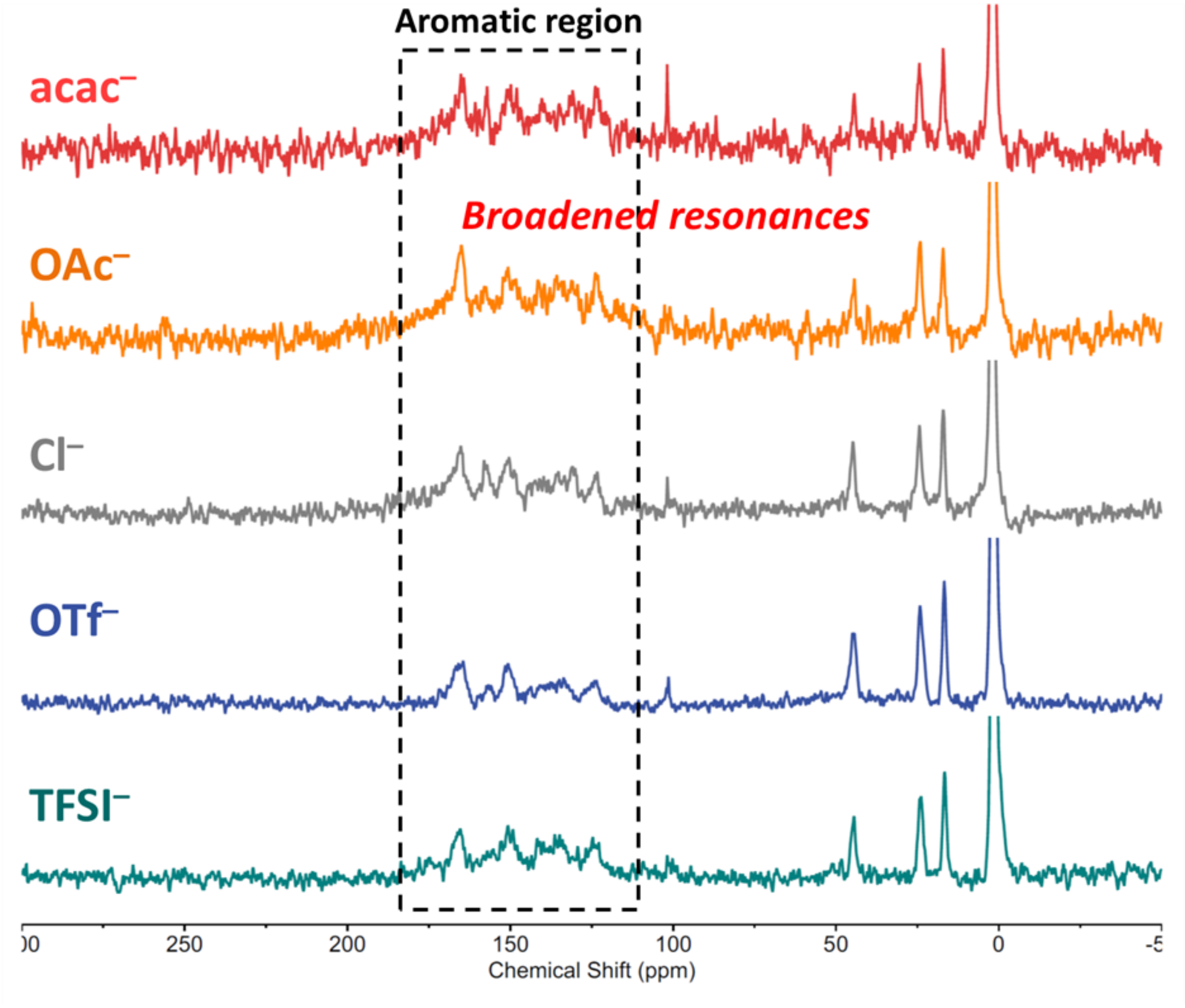


**Supplementary Fig. 8 | Solid state NMR analysis of Zn-X-BPy-PDMS polymers.** $^{13}C$ CP/MAS solid state NMR (100 MHz) spectra of Zn-X-BPy-PDMS polymers. Broadening of aromatic BPy carbon resonances (in black dashed box) were detected in Zn-acac-BPy-PDMS and Zn-OAc-BPy-PDMS.

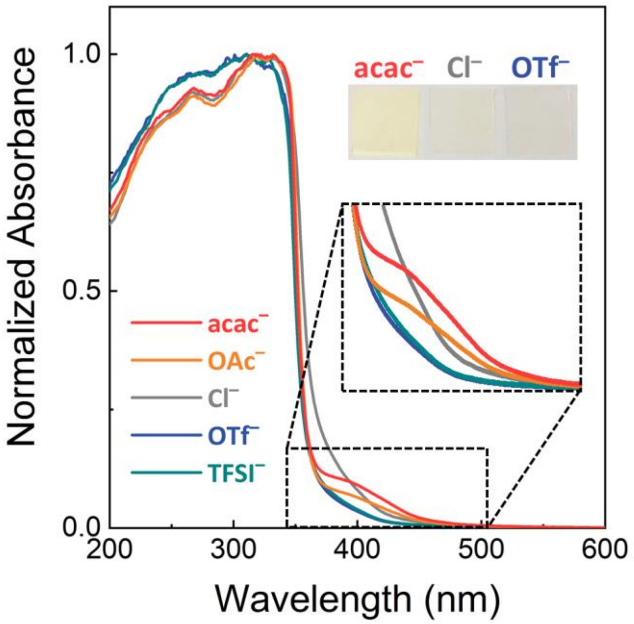


**Supplementary Fig. 9 | Film UV-vis studies on Zn-X-BPy-PDMS polymers.** UV-vis absorption spectra of Zn-X-BPy-PDMS polymer films ($T$ = 298 K). The spectra for the absorption at a 350–500 nm region are highlighted with the black dashed box. Multimodal anions exhibit longer wavelength absorption band exclusively. Inset: photographic images of Zn-acac-BPy-PDMS, Zn-Cl-BPy-PDMS, and Zn-OTf-BPy-PDMS films.

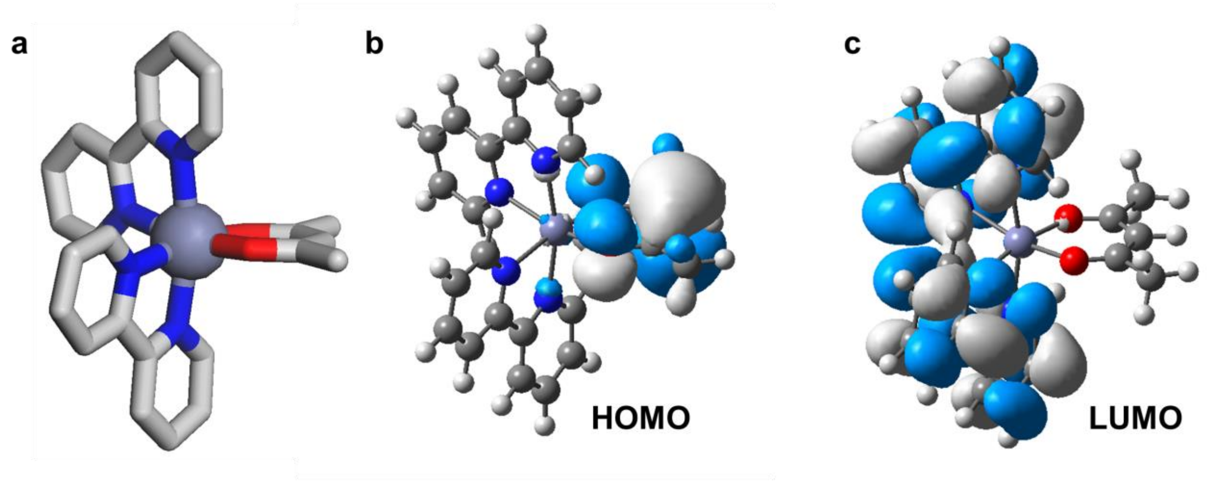


**Supplementary Fig. 10 | DFT calculation on $[Zn(BPy)_2(acac)]^+$ complex.** **a**, DFT (B3LYP/6-31++G(d,p)) energy-minimized structure of $[Zn(BPy)_2(acac)]^+$ complex, and its **b**, HOMO which is localized in acac$^-$, and **c**, LUMO which is localized in BPy.

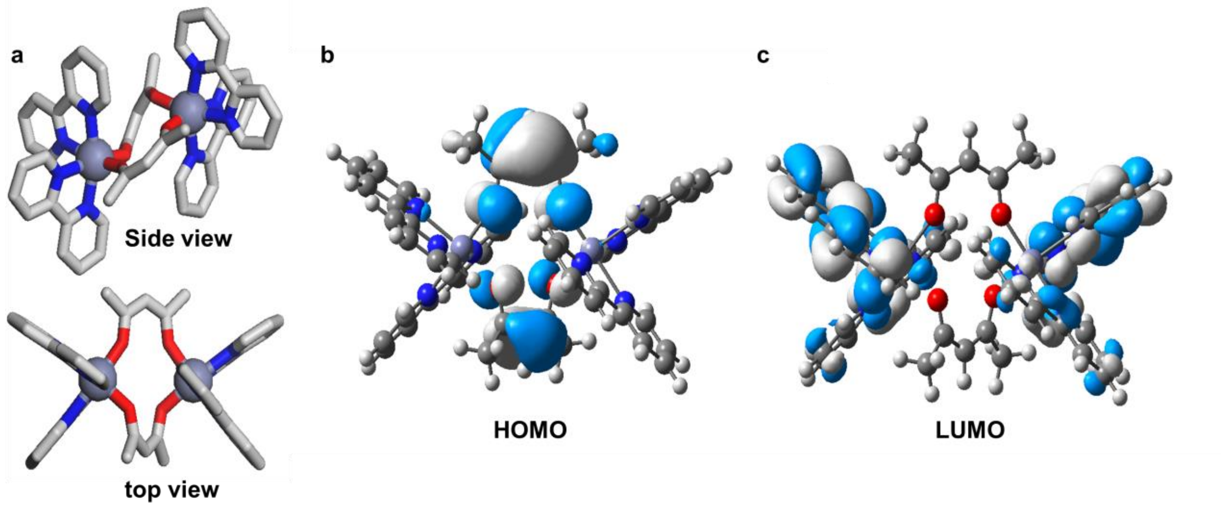


**Supplementary Fig. 11 | DFT calculation on acac$^-$-bridged bimetallic zinc complex.** **a**, DFT (B3LYP/6-31++G(d,p)) energy-minimized structure of the acac$^-$-bridged bimetallic zinc complex, and its **b**, HOMO which is localized in bridging acac$^-$, and **c**, LUMO which is localized in BPy.

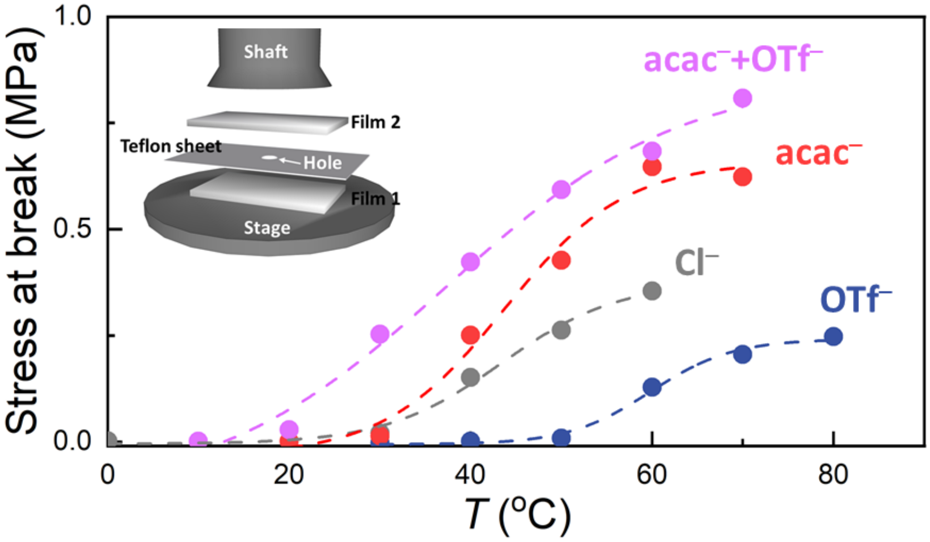


**Supplementary Fig. 12 | Self-healing tests.** Temperature-dependent self-healing behaviour of Zn-X-BPy-PDMS films ($acac^-$; red, $Cl^-$; gray, $OTf^-$; blue, $acac^-$+$OTf^-$; pink). Inset: schematic illustration of the experimental set-up.

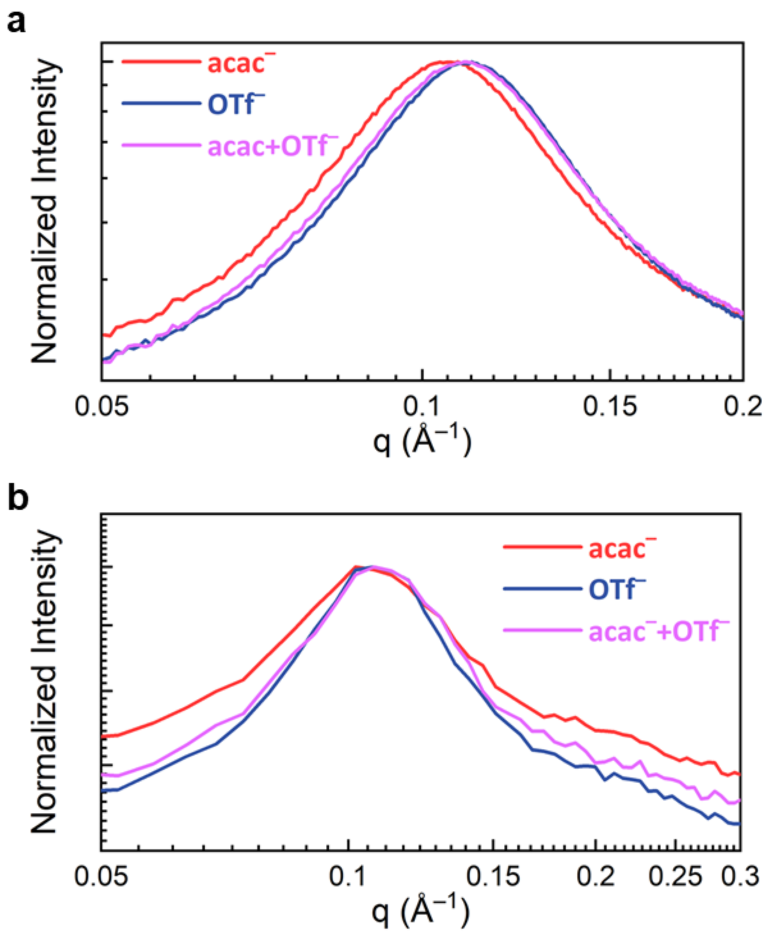


**Supplementary Fig. 13 | SAXS and SANS analysis of Zn-acac-OTf-BPy-PDMS.** Film SAXS (**a**) and SANS (**b**) profiles of Zn-acac-BPy-PDMS (red), Zn-OTf-BPy-PDMS (blue), and Zn-acac-OTf-BPy-PDMS in a 1:1 ratio (pink). The scattering profile of Zn-acac-OTf-BPy-PDMS is almost superimposable with that of Zn-OTf-BPy-PDMS for both SAXS and SANS measurements.

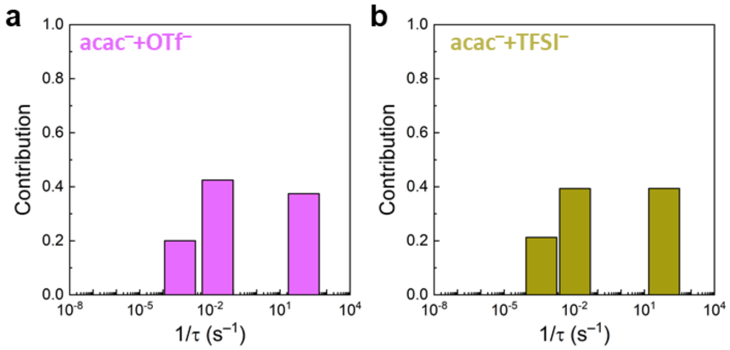


**Supplementary Fig. 14 | Contributions of each relaxation component to overall energy dissipation of Zn-acac-OTf- and Zn-acac-TFSI-BPy-PDMS polymers.** Relative contributions of each relaxation component of **a**, Zn-acac-OTf-BPy-PDMS, and **b**, Zn-acac-TFSI-BPy-PDMS. The sum of the three contributions equals to 1 for all the polymers.

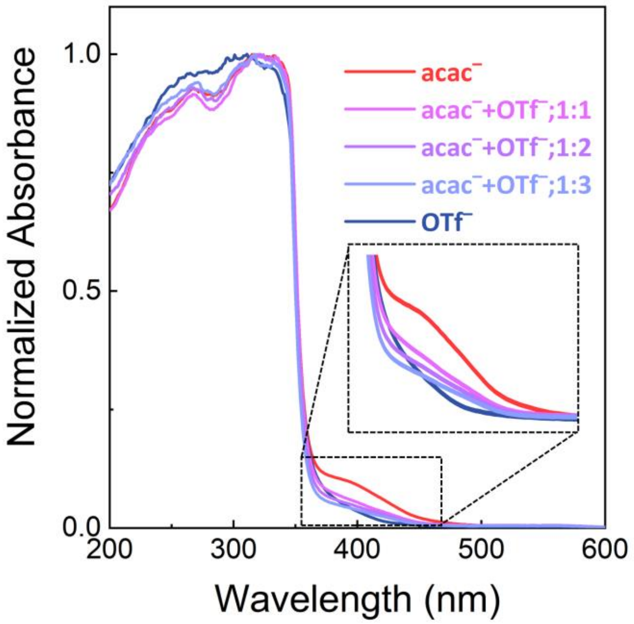


**Supplementary Fig. 15 | Film UV-vis studies on Zn-acac-OTf-BPy-PDMS polymers.** UV-vis absorption spectra of Zn-acac-OTf-BPy-PDMS polymer films ($T$ = 298 K) with varying molar ratio of acac⁻ and OTf⁻. The spectra for the absorption at a 350–450 nm region are highlighted with the black dashed box.

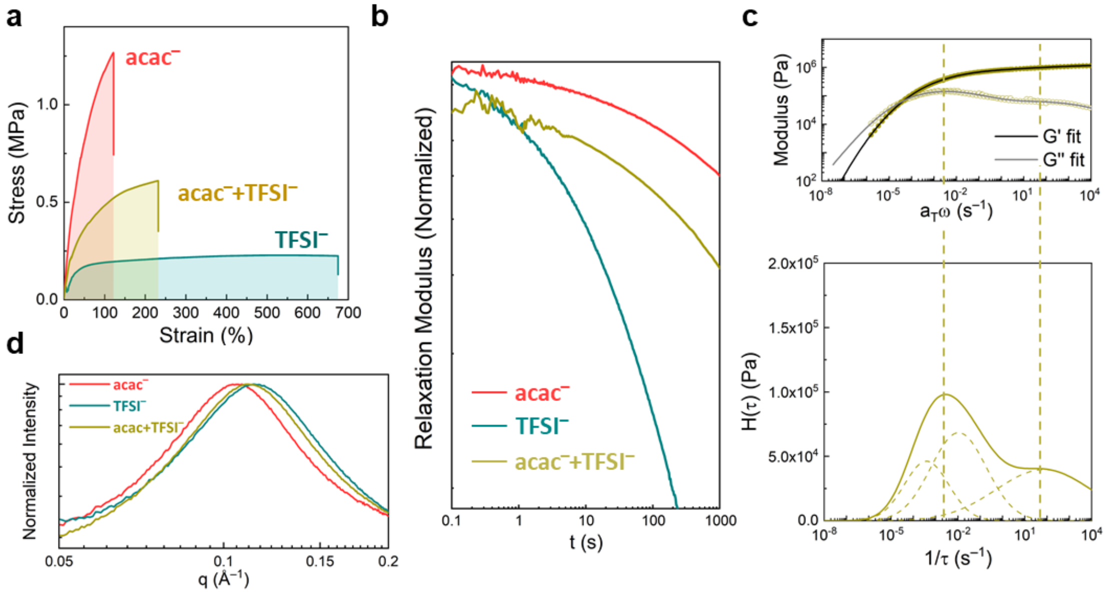


**Supplementary Fig. 16 | Mechanical and dynamic properties of Zn-acac-TFSI-BPy-PDMS polymer.** **a**, Stress–strain curves of polymer films comprising $acac^-$ (red), $TFSI^-$ (green), and a 1:1 mixture of $acac^-$ and $TFSI^-$ (yellow), showing no synergistic effect of anions (a sample width of 3 mm, a thickness of 0.2–0.4 mm, and a length of 5 mm at a loading rate of 5 mm $min^{-1}$). **b**, Shear stress relaxation spectra of polymer films under a shear strain of 3%. The relaxation spectrum of Zn-acac-TFSI-BPy-PDMS appears in the middle of the spectra of Zn-acac-BPy-PDMS and Zn-TFSI-BPy-PDMS. **c, top**, Experimental master curve (yellow dots) and best-fit lines (G'; black line, G''; gray line) of Zn-acac-TFSI-BPy-PDMS. **c, bottom**, Relaxation time spectrum, H($\tau$) of Zn-acac-TFSI-BPy-PDMS which exhibits $acac^-$-like broad and multiple peaks. The dashed lines in the plot indicate the spectra of each energy dissipation mode. **d**, Film SAXS profiles of Zn-acac-BPy-PDMS (red), Zn-TFSI-BPy-PDMS (green), and Zn-acac-TFSI-BPy-PDMS (yellow). The scattering profile of Zn-acac-TFSI-BPy-PDMS appears in the middle of the profiles of Zn-acac-BPy-PDMS and Zn-TFSI-BPy-PDMS.

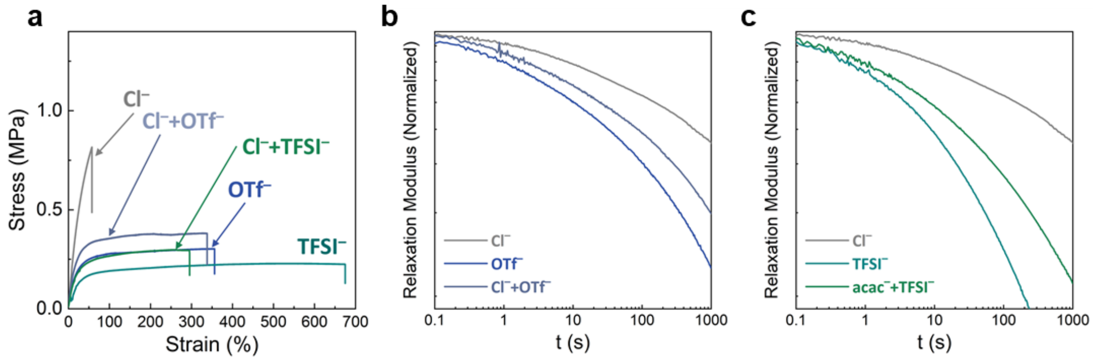


**Supplementary Fig. 17 | Mechanical and dynamic properties of Zn-Cl-OTf- and Zn-Cl-TFSI-BPy-PDMS polymers. a**, Stress–strain curves of Zn-Cl-BPy-PDMS (grey), Zn-OTf-BPy-PDMS (blue), Zn-TFSI-BPy-PDMS (green) films and their mixed combinations, Zn-Cl-OTf-BPy-PDMS (blue-grey), Zn-Cl-TFSI-BPy-PDMS (light-green) films (a sample width of 3 mm, a thickness of 0.2–0.4 mm, and a length of 5 mm at a loading rate of 5 mm $min^{-1}$). **b**, **c**, Shear stress relaxation spectra of polymer films under a shear strain of 3%. In both cases, the relaxation spectrum of the mixed anions appears in the middle of the spectra of the single anion system.

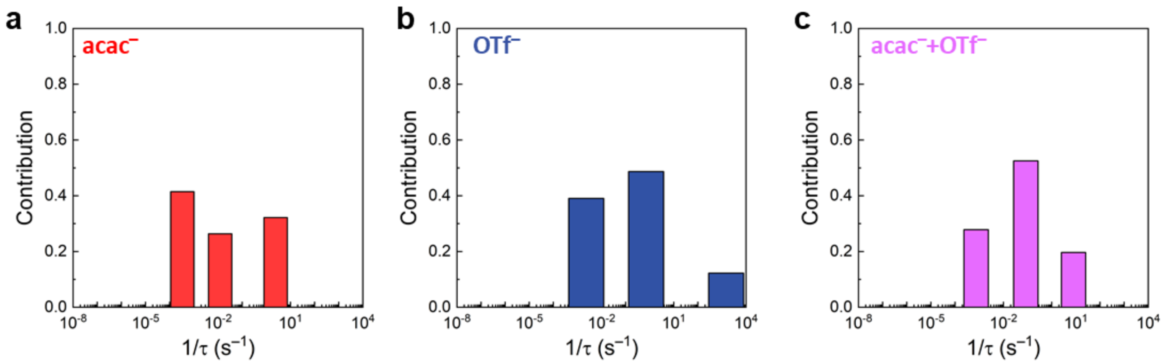


**Supplementary Fig. 18 | Contributions of each relaxation component to overall energy dissipation of Cu-X-BPy-PDMS polymers.** Relative contributions of each relaxation component of **a**, Cu-acac-BPy-PDMS, and **b**, Cu-OTf-BPy-PDMS, and **c**, Cu-acac-OTf-BPy-PDMS. The sum of the three contributions equals to 1 for all the polymers.

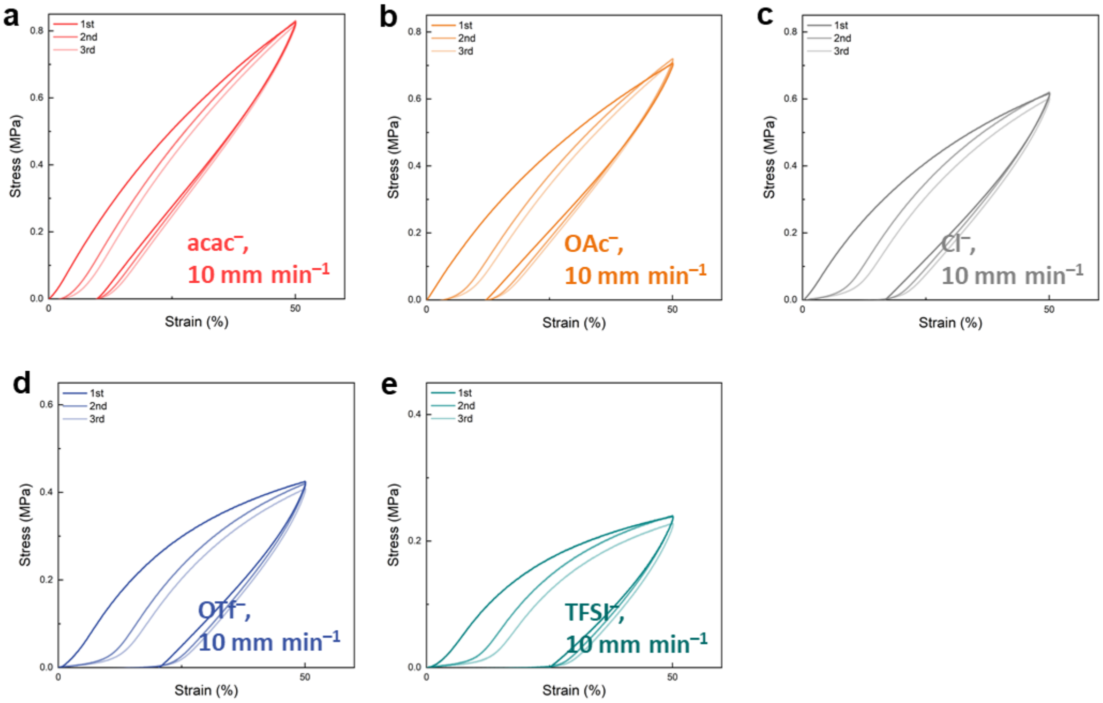


**Supplementary Fig. 19 | Cyclic loading–unloading tests.** Stress–strain curves of the polymer films with a sample width of 3 mm, a thickness of 0.2–0.4 mm, and a length of 5 mm under cyclic loading at loading rates of 10 mm $min^{-1}$. Three cycles with a time interval of 30 min were performed for each measurement. **a**, $acac^{-}$, **b**, $OAc^{-}$, **c**, $Cl^{-}$, **d**, $OTf^{-}$, **e**, $TFSI^{-}$.

## Supplementary Tables

| Entry | Counter anion | Young's modulus[a] (MPa) | Tensile strength (MPa) | Fracture strain (%) | Mechanical Toughness (x $10^6$ J $m^{-3}$) |
|---|---|---|---|---|---|
| 1 | $acac^-$ | 2.9 | 1.27 | 109 | 1.02 |
| 2 | $OAc^-$ | 1.7 | 0.73 | 96 | 0.45 |
| 3 | $Cl^-$ | 1.4 | 0.53 | 65 | 0.22 |
| 4 | $OTf^-$ | 1.3 | 0.30 | 356 | 0.97 |
| 5 | $TFSI^-$ | 0.86 | 0.22 | 675 | 1.41 |

**Supplementary Table 1 | Summary of the mechanical properties of Zn-X-BPy-PDMS polymers.** Mechanical properties of Zn-X-BPy-PDMS polymer films under a loading rate of 100% $min^{-1}$. [a] calculated from the initial slope of stress–strain curves (within 5%).

| Entry | Counter anion | van der Waals volume (Å$^3$) | van der Waals surface area (Å$^2$) |
|---|---|---|---|
| 1 | acac$^-$ | 93.56 | 124.89 |
| 2 | OAc$^-$ | 55.31 | 75.34 |
| 3 | Cl$^-$ | 20.41 | 36.46 |
| 4 | OTf$^-$ | 81.92 | 107.36 |
| 5 | TFSI$^-$ | 151.56 | 181.25 |

**Supplementary Table 2 | DFT-calculated size of the counter anions.** van der Waals volumes and surface areas of the counter anions calculated from DFT (B3LYP/6-31G(d)) energy-minimized structures.

## Supplementary References


1. Rao, Y.-L. *et al.* Stretchable Self-Healing Polymeric Dielectrics Cross-Linked Through Metal-Ligand Coordination. *J. Am. Chem. Soc.* **138**, 6020–6027 (2016).
2. Rao, Y.-L., Feig, V., Gu, X., Wang, G.-J. N. & Bao, Z. The effects of counter anions on the dynamic mechanical response in polymer networks crosslinked by metal-ligand coordination. *J. Polym. Sci. A Polym. Chem.* **55**, 3110–3116 (2017).
